# Prevalence of anaemia in older persons: systematic review

**DOI:** 10.1186/1471-2318-8-1

**Published:** 2008-01-14

**Authors:** Helen Gaskell, Sheena Derry, R Andrew Moore, Henry J McQuay

**Affiliations:** 1 Pain Research, Nuffield Department of Anaesthetics, University of Oxford, Oxford Radcliffe Hospitals, The Churchill, Headington, Oxford, OX3 7LJ, UK

## Abstract

**Background:**

Ageing populations will impact on healthcare provision, especially since extra years are not necessarily spent in good health. It is important to identify and understand the significance of common medical problems in older people. Anaemia may be one such problem. We report on the prevalence of anaemia in cohorts of elderly people in the general population. The presence of anaemia is associated with a worse prognosis for both morbidity and mortality.

**Methods:**

Electronic searching and reference lists of published reports were used to identify studies that reported on prevalence of anaemia in cohorts of at least 100 individuals predominantly aged 65 years and over living in developed countries, together with criteria used to define anaemia. Studies of anaemia prevalence in specific disease groups or published before 1980 were excluded. Prevalence data for the entire cohort, for men and women separately and for different age bands were extracted.

**Results:**

Forty-five studies contributed data. Thirty-four studies (n = 85,409) used WHO criteria to define anaemia. The weighted mean prevalence was 17% (3–50%) overall, and 12% (3–25%) in studies based in the community (27, n = 69,975), 47% (31–50%) in nursing homes (3, n = 1481), and 40% (40–72%) in hospital admissions (4, n = 13,953). Anaemia prevalence increased with age, was slightly higher in men than women, and was higher in black people than white. Most individuals classified as anaemic using WHO criteria were only mildly anaemic.

**Conclusion:**

Anaemia, as defined by WHO criteria, is common in older people living in the community and particularly common in nursing home residents and hospital admissions. Predicted demographic changes underline the need to understand more about anaemia in older people.

## Background

The world population is ageing; in 2000 there were 600 million people aged 60 or over, and it is estimated that this figure will double by 2025, and more than triple to 2 billion by 2050 [1]. Between 2007 and 2027 the number of people aged 65 years or older in England alone is predicted to increase by 200,000 each year, rising from eight to 12 million over that 20-year period [2]. In the USA numbers aged 65 years or older are predicted to rise from 37 million in 2006 to 63 million by 2025 [3].

The "oldest old", often defined as those aged 85 or older, show the largest increase in numbers. By 2066 it is anticipated that there will be almost a third of a million people aged 100 years or older, in a population of 70 million in the UK [2]. In the USA the prediction is for a third of a million over the age of 100 years by 2020 [3].

This demographic change has obvious implications for individuals and society. It will impact particularly on healthcare provision, especially as longer life does not necessarily mean more years enjoyed in good health. It becomes increasingly important to highlight common medical problems in older people, especially if their extent and potential significance are not generally recognised. We suspected that anaemia might be one such problem.

Anaemia is often defined in terms of the WHO criteria, established in 1968 [4]. The WHO definition of anaemia is a haemoglobin (Hb) concentration <130 g/L in men, and <120 g/L in women. There has been debate about the use of these values [5], and in particular, whether they should be used to define anaemia in older people [6], but there is no widely accepted alternative definition of anaemia in this age group.

In general, haemoglobin levels are lower in older than in younger people. The reasons for this are not completely understood. It is unclear whether haemoglobin falls in older people because this is a feature of normal ageing, or whether it is always pathological, even if underlying conditions cannot be identified. In an individual patient it may be that some decline in haemoglobin occurs as part of normal ageing, but that disease may also contribute to the development of anaemia.

Anaemia of chronic disease, or anaemia of chronic inflammation, is the term associated with some chronic medical conditions, such as chronic renal disease and rheumatoid arthritis. Older people who suffer such chronic conditions might be expected to be anaemic, just as younger patients are. However, it is not always appreciated that older patients who suffer other medical problems, for example cognitive impairment, have a worse prognosis if they are also anaemic. It is not surprising that anaemia is associated with heart failure or myocardial infarction or death, but the reasons underlying the association between anaemia and other morbidity are less tangible.

We report on the prevalence of anaemia in cohorts of older people in the general population.

## Methods

MEDLINE and EMBASE (Ovid interface) were searched for the years 1980 to February 2007 for studies reporting prevalence of anaemia in elderly populations or cohorts, using search strategies described in Additional file 1. It is known that observational studies can be difficult to identify through electronic searching [7,8], so reference lists of retrieved studies and reviews were also searched carefully for further reports, together with "linked articles" in PubMed. Full reports were obtained whenever possible for all studies identified from their abstracts as potentially useful.

Studies were included if they reported prevalence of anaemia for cohorts of at least 100 individuals aged predominantly 65 and over living in developed countries, together with the criteria used to define anaemia. Studies of anaemia prevalence in specific disease groups (diabetes, rheumatoid arthritis, renal disease) or in "extreme" populations (extreme poverty, poor nutrition) were excluded. Where more than one paper reported on the same or overlapping cohorts, or subgroups of large cohorts, care was taken to avoid double counting, and the report with the most information on the largest number of individuals was used. We limited the search to studies published since 1980 to avoid possible historical changes in prevalence, which might be due to improvements in diet and general health.

For each report we extracted information on the country of residence, how the cohort was selected (for instance, random sampling, inclusive cohort) and whether individuals were living in the community, in nursing homes or were hospitalised, together with numbers studied, sex, mean age, criteria used to define anaemia, comorbidities and specific inclusion or exclusion criteria. For anaemia we sought details of overall prevalence, prevalence by age group, and prevalence in men and women separately. Where associations were found between anaemia and particular outcomes (mortality, cognitive function, disability) or medication use, these were also noted.

Two authors (HG, SD) carried out searching, study selection, and data extraction independently and disagreements were resolved by consensus (RAM, HG, SD).

It was recognised that the studies would be heterogeneous both in terms of the cohorts and the criteria used to define anaemia, so that any planned analysis should be simple and in large part qualitative. We did plan to calculate a weighted mean prevalence using the most commonly used definition of anaemia both overall, and for men and women separately. Sensitivity analyses were planned for community-living versus institutionalised (nursing home) or patients admitted to hospital, and for older (1980–1999) versus recent (2000–2006) studies. The weighted mean was calculated by dividing the sum of the product of prevalence (as percentage) and number of patients by the sum of the number of patients.

## Results

Electronic searches identified a total of 293 and 153 articles in Medline and EMBASE respectively, and many more from bibliographies of retrieved papers or "linked articles" in PubMed. A total of 83 studies were reviewed in detail, and 45 [9-53] were included, with details in Additional file 2. For two included studies [35,40] the full paper was not available, but adequate details were given in the abstract, and another [25] was published as a letter rather than a full report. A list of excluded studies, with reason for exclusion, is in Additional file 3.

Most studies were of moderate size. The distribution of the 35 studies using WHO criteria of anaemia by size is shown in Table 1. Only two examined at least 10,000 individuals ([13,16] Table 1), and the five studies with at least 5,000 individuals contributed over 60% of the total number of individuals. Twenty-six studies (76% of the total studies) examined fewer than 2,500 individuals (25% of total individuals). Almost half the 35 studies had fewer than 1,000 individuals, and contributed less than 8% of the total.

**Table 1:** Studies reporting anaemia in older persons using WHO criteria

	**Number of**		**Percent of total**	
	
**Size range (individuals)**	**Studies**	**Patients**	**Studies**	**Patients**
10,000–20,000	2	30,331	5.9	35.5
5,000–10,000	3	21,590	8.8	25.3
2,500–5,000	3	12,373	8.8	14.5
1,000–2,500	10	14,867	29.4	17.4
100–1,000	16	6,248	47.1	7.3

The majority (34/45, 76%) of studies were of individuals living independently in the community, although a few included some individuals in nursing homes or hospital. Four studies [17,22,23,40] were in nursing homes and seven studies [12,14,16,25,30,37,41] assessed patients who had been admitted to hospital.

The WHO criteria of Hb < 130 g/L for men and <120 g/L for women was by far the most commonly used to define anaemia, though we did accept results from three studies [15,19,43] that used thresholds within 5 g/L of these. Other criteria included Hb < 140 g/L for men and 120 g/L for women, Hb levels of <120 to <80 g/L for both sexes, or various percentiles of the cohort. Haematocrit (Hct) levels are sometimes used to define anaemia. For example Hct 39% for men and 36% for women are alternative WHO criteria for defining anaemia [4], and two studies using Hct were included in the analysis of studies using WHO criteria (± 1%) [29,52]. Where studies used a much lower concentration of haemoglobin to define anaemia, for instance <120 g/L or <110 g/L, we also extracted the prevalence information for these lower levels.

**Figure 1:**
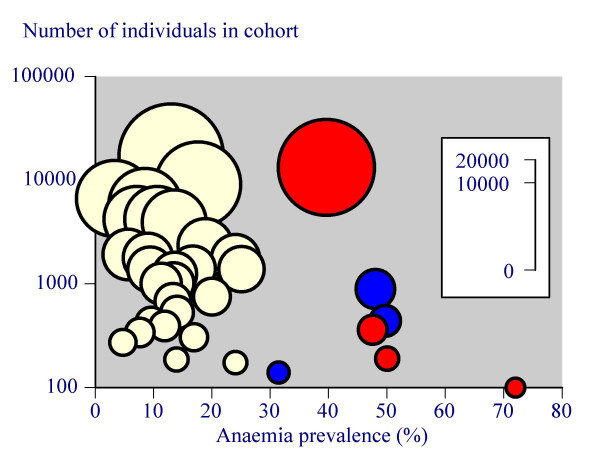
Anaemia prevalence according to the size of cohort (non-linear inset scale). Yellow symbols represent older people living in the community, blue those in nursing homes, and red admission to hospital.

Thirty-four studies, with 85,409 individuals, using WHO criteria, yielded a weighted mean anaemia prevalence of 17% (range 3% to 50%). The weighted mean prevalence was 12% (3% to 26%) in 27 studies predominantly in the community (69,975 individuals), 47% (31% to 50%) in three studies in nursing homes (1481 individuals [17,22,53]) and 40% (40% to 72%) in four studies in hospital admissions (13,953 individuals [12,14,16,25]; Table 2). Figure 1 shows the distribution of anaemia prevalence according to population (community, nursing home, and hospital admission) and cohort size. Anaemia was more prevalent in nursing homes or hospital admissions than in the community, and there was no overlap in anaemia prevalence between these groups (Figure 1).

**Table 2:** Summary of results, including sensitivity analyses and use of alternative criteria for anaemia

	**Number of studies**	**Number of individuals**	**Anaemia (weighted mean %)**
	
**WHO criteria**			
			
All studies	34	85409	17
Community/Nursing Home	31	71456	13
Community only	27	69975	12
Nursing Homes only	4	1481	47
Hospital admissions only	3	13953	40
			
**Sensitivity analyses**			
			
Community/Nursing Home before 2000	12	19708	15
Community/Nursing Home 2000 and later	18	51748	12
Men	26	23856	15
Women	28	35781	14
Community only using Hb only	26	68912	12
			
**Alternative criteria**			
			
Community/Nursing Home Hb ≤ 120 g/L	3	2516	9
Community/Nursing Home Hb ≤ 110 g/L	4	21611	6

Note: Individual studies were weighted by number of individuals for calculation of overall mean rate of anaemia

Two studies in the community (2341 individuals) [39,51], and one in a nursing home (175 individuals) [40] defined anaemia as Hb ≤ 120 g/L, giving prevalence rates of 7%, 11% (weighted mean 8%) and 24% respectively. A further three studies (21,170 individuals) [35,51,10] in the community and one in a nursing home (441 individuals) [17] used Hb ≤ 110 g/L, giving prevalence rates of 3%, 13%, 3% (weighted mean 6%) and 25% respectively. Generally, prevalence fell rapidly with lower thresholds, indicating that the vast majority of individuals classified as anaemic using WHO criteria were in fact only mildly anaemic.

**Figure 2:**
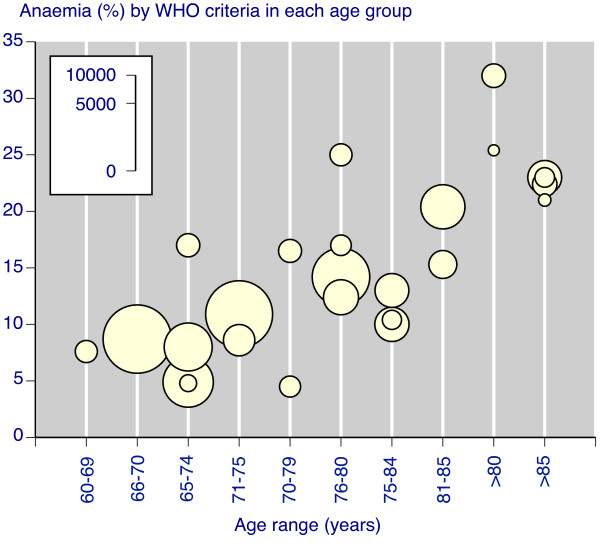
Anaemia prevalence by age range. Size of the symbol is proportional to the size of the cohort (inset scale)

Sensitivity analyses (Table 2) examining the effect of publication date in the community or nursing home studies found similar prevalence in those published before 2000 (15%) and since 2000 (12%). Prevalence was slightly higher in men (15%) than women (14%) in studies reporting by sex. The two studies using Hct to define anaemia contributed less than 3% of the total population in community studies and excluding them did not alter the weighted mean estimate. Eight studies reported anaemia prevalence in different age groups, and there was a clear increase in prevalence with age (Figure 2). Other studies reported an increase in prevalence with age, but without giving the numbers.

Five studies commented on prevalence of anaemia and ethnicity [52,11,21,24,42]. All indicated that anaemia prevalence was higher in black populations than white and Mexican-Americans, by a factor of about three times.

## Discussion

These results confirm that anaemia, as defined by the WHO criteria, is common in older people, affecting 1 in every 7 or 8 over-65s living in the community. Even using more stringent definitions of haemoglobin less than 120 g/L or 110 g/L, anaemia affects 1 in 12 to 1 in 15 older people in the community. In nursing homes, or in older people admitted to hospital, anaemia will be even more common, affecting almost 1 in 2. Higher prevalence occurs at greater age, and in black people.

These broad assertions have limitations. Firstly we have chosen to use the WHO criteria for defining anaemia because they are the most commonly reported. Threshold values are to some extent arbitrary, and WHO values are in the range of haemoglobin levels where the number of individuals captured changes rapidly. Choosing slightly different thresholds can considerably alter the number defined as anaemic. While we recognise the limitations of using thresholds, we have used WHO criteria because that is what is reported in the literature and because they do appear to be relevant, correlating with clinical outcomes.

We know, furthermore, that electronic searching for observational studies identifies fewer than half of studies, so that other methods have to be employed [7,8]. While we examined bibliographies extensively, it is likely that some information was not identified by our searches. Though we limited our inclusion criteria to studies with at least 100 individuals, Table 1 demonstrates that small studies contributed little to the overall numbers, which were dominated by five studies of more than 5,000 individuals. Overall there was information on prevalence for almost 86,000 individuals, over 60% of whom were in these large studies. This justifies excluding prevalence studies with fewer than 100 subjects; non-inclusion of even 100 trials with fewer than 100 subjects would have contributed a maximum of 10% more to the total, and we found that 50% of the studies included with the smallest numbers actually contributed 8% to the total.

Only 34 of 45 studies used the WHO criteria of anaemia established in 1968 [4]. With highly variable definitions and prevalence clearly influenced by definition, we concentrated on the studies using the WHO definition. Additionally we arbitrarily imposed a 1980 cut off for publications to restrict influences from very different times from today. A sensitivity analysis comparing studies published between 1980 and 2000 and those after 2000 did not show any large temporal variation, though (Table 2).

Even the studies we accessed did not all have the determination of anaemia prevalence in older people as their prime outcome. Rather, they were studies of association of anaemia with morbidity or disease, or examined anaemia as only one element of the study. Moreover, there were very limited amounts of information on important subgroups, like the oldest old, those with comorbities, or with ethnic or cultural differences. Information might be indicative, but it is difficult to have confidence in the precision of estimates in subgroups with such small numbers. More detailed studies can help, as with a recent study on racial variation and anaemia mortality and morbidity [54].

A degree of heterogeneity in the cohort studies was expected, and was found. We chose a crude separation between individuals living in the community, in nursing homes, or admitted to hospital as obvious differences between individuals, but within each cohort different distributions of age, ethnicity, and conditions would apply. Few studies tried, for instance, to give a population-based assessment by sampling according to a population norm. Sampling methods varied greatly. Some cohorts were inclusive, while others used random sampling, sometimes stratified, or with deliberate over-sampling of minority groups (ethnic, older old), and others did not specify methods.

Two very large studies could not be included because they did not define anaemia. The larger reported on over 1.1 million individuals in a general Medicare database [55], and reported patients with ICD-9-CM coding of anaemia rather than any particular threshold of haemoglobin or haematocrit. The diagnosis of anaemia was made in 15% of individuals, with a mean age of 77 years. Another large study of 43,510 nursing home residents reported 19% prevalence of anaemia, but without stating the criteria used [56]. Both were largely in line with the results of this review.

Another very large study of 91,000 patients with recent myocardial infarction could not be included because it used Hct of ≤ 39% as the threshold for anaemia in both men and women [57]. This study did give a breakdown by different haematocrit bands and sex, but reported a denominator from which individuals with high haematocrit values were excluded. Adding back these individuals, and assuming the same sex distribution amongst them as in the next highest band, gave an overall prevalence of 26% (28% in men, 25% in women). This is lower than the included studies of hospital admissions, but is difficult to interpret because of the clinical characteristics of the patients and the assumptions that had to be made to calculate prevalence.

A previous systematic review [58] included 71 studies on patients aged 60 years or older, but limited analysis to effects of sex differences reported in nine studies, and age differences reported in six studies. This new review used searches conduced about six years later, with wider inclusion criteria (such as abstracts), but more stringent criteria (such as definition of anaemia using WHO criteria, and exclusion of small studies). A consequence was that we reviewed a smaller number of studies, probably of higher quality, and examined issues not examined previously, such as the link between setting and anaemia prevalence.

The previous review concluded that anaemia prevalence was generally higher in men than women. We found only a small difference, but confined our comparison to studies using the same WHO criteria for anaemia, whereas Beghe et al [58] included studies using different definitions, including thresholds for men ranging from 110 g/L to 140 g/L. The finding of increasing prevalence with older age was confirmed in our analysis, though with more studies (Figure 2); we did not include one small report of anaemia prevalence of 53% in 89 centenarians [59]. This review, limited to persons aged 65 years and older, and to studies of at least 100 patients, additionally points to differences in anaemia prevalence between older people living in the community, in nursing homes, or admitted to hospital. The previous review [58] also noted the difficulties in drawing meaningful conclusions from disparate studies, and while we did extract potentially useful information from the studies (Additional file 2), we felt that clinical and methodological heterogeneity, and disparities in size, were too great to attempt further sensitivity analysis.

The rapid fall in prevalence of anaemia with definitions using lower thresholds (such as 110 g/L) suggests that in most cases the anaemia is mild. Despite this, the presence of anaemia in older people is associated with poorer outcomes. Older people (mean age 77 years) with anaemia suffered higher mortality (57%) than those without anaemia (39%) when followed up for almost 12 years [21]. Both anaemia and renal insufficiency appear to be independent risk factors for death among patients with heart failure [59] and after myocardial infarction [60], and anaemia predicts mortality in severe heart failure [61,62]. Anaemia is also associated with increased risk of falls and impaired muscle strength [63], executive function impairment [64] and dementia [65], hospital admission [66], and longer hospital stay [50,66].

Knowing the prevalence of anaemia in older people is important because we can begin to estimate the extent of the problem in this group. In the UK in 2007 there are almost 10 million people aged 65 years or older; the implication is that 1.5 million have anaemia as defined by WHO, and perhaps 600,000 have haemoglobin levels below 110 g/L. In thirty years there will probably be half as many again.

Studies have consistently demonstrated an approximately three times higher prevalence in black people between black and white people [5,67] of all ages, not confined to the elderly, though differences narrowed when iron-deficient or thalassaemic patients were excluded [67]. While the reasons and consequences may be uncertain, and this is an area of some controversy, the use of different diagnostic criteria is not, so far, suggested, though attempts have been made to do so [5]. The literature we reviewed provided little information about the prevalence and consequences of the racial differences in anaemia. As has been pointed out [5], the genes of individuals classified as African-American could be anywhere between 10% and 90% of European origin. There is, in addition, a gradient in haemoglobin concentrations between whites of northern versus southern European ancestry [5]. This is clearly not a simple topic.

The similar prevalence found in studies published before and after 2000 suggests that significant advances in identifying and/or treating anaemia in older people have not been made in recent years. It is estimated that for between 14% and 50% of cases of anaemia in the elderly, the underlying cause is unexplained [24,57]. We know little about whether it is preventable, or how much is associated with medications for other medical conditions of older age, except for NSAIDs [68] and perhaps low dose aspirin [13]. There does not appear to be an extensive literature on therapy, and whether therapy improves quality of life and outcomes in older people.

We, as others before [58], find the limitations of the literature, especially the small number of larger studies in defined communities with defined criteria, limit any methodological considerations without the danger of over analysis. To some extent the unanswered questions are more interesting than those that can be answered – for example about the causes, health and economic consequences, and treatment of anaemia in older people, and the desirability or otherwise of using different action levels than the WHO definitions of anaemia. While the usual call for more research is often rather lame, in this case the extraordinary expected growth in the numbers of older people, coupled with the high prevalence and potentially serious consequences of anaemia, suggest that in this case more research is a sensible conclusion.

## Conclusion

Anaemia as defined using WHO criteria is common in older people living in the community, and is higher in the older old and black people. It is particularly common amongst nursing home residents and older people admitted to hospital. The presence of anaemia is associated with a worse prognosis, with increases in morbidity and mortality. Predicted demographic changes will increase the burden and underline the need to understand more about anaemia in older people.

## Abbreviations

WHO World Health Organisation

Hb haemoglobin

Hct haematocrit

NSAID non-steroidal anti-inflammatory drug

## Competing interests

RAM & HJM have received lecture fees from pharmaceutical companies. The authors have received research support from charities and government sources at various times.

## Authors' contributions

HG and SD were involved with searching, data extraction, analysis and writing the manuscript; RAM and HJM with planning the study, analysis, and writing. All authors read and approved the final manuscript.

## Pre-publication history

The pre-publication history for this paper can be accessed here:



## Supplementary Material

Additional file 1 Electronic search strategy Click here for file

Additional file 2 Included studies. Details of studies included in the review Click here for file

Additional file 3 Excluded studies. Citation and reason for exclusion Click here for file

## References

[B1] World Health Organization, ageing and life course. http://www.who.int/ageing/en.

[B2] Government Actuary's Department. http://www.gad.gov.uk.

[B3] U.S. Census Bureau. http://www.census.gov.

[B4] World Health Organisation (1968). Nutritional anemia: report of a WHO Scientific Group.

[B5] Beutler E, Waalen J (2006). The definition of anaemia: what *is *the lower limit of normal of the blood hemoglobin concentration?. Blood.

[B6] Nilsson-Ehle H, Jagenburg R, Landahl S, Svanborg A (2000). Blood haemoglobin declines in the elderly: implications for reference intervals from age 70 to 88. Eur J Haematol.

[B7] Lemeshow AR, Blum RE, Berlin JA, Stoto MA, Colditz GA (2005). Searching one or two databases was insufficient for meta-analysis of observational studies. J Clin Epidemiol.

[B8] Ruppen W, Derry S, McQuay H, Moore RA (2006). Incidence of epidural hematoma, infection, and neurologic injury in obstetric patients with epidural analgesia/anesthesia. Anesthesiology.

[B9] Atti AR, Palmer K, Volpato S, Zuliani G, Winblad B, Fratiglioni L (2006). Anaemia increases the risk of dementia in cognitively intact elderly. Neurobiol Aging.

[B10] Culleton BF, Manns BJ, Zhang J, Tonelli M, Klarenbach S, Hemmelgarn BR (2006). Impact of anemia on hospitalization and mortality in older adults. Blood.

[B11] Denny SD, Kuchibhatla MN, Cohen HJ (2006). Impact of anemia on mortality, cognition, and function in community-dwelling elderly. Am J Med.

[B12] Dharmarajan TS, Avula S, Norkus EP (2006). Anemia increases risk for falls in hospitalized older adults: an evaluation of falls in 362 hospitalized, ambulatory, long-term care, and community patients. J Am Med Dir Assoc.

[B13] Hammerman-Rozenberg R, Jacobs JM, Azoulay D, Stessman J (2006). Aspirin prophylaxis and the prevalence of anaemia. Age Ageing.

[B14] Joosten E, Lemiengre J, Nelis T, Verbeke G, Milisen K (2006). Is anaemia a risk factor for delirium in an acute geriatric population?. Gerontology.

[B15] Loikas S, Koskinen P, Irjala K, Lopponen M, Isoaho R, Kivela SL, Pelliniemi TT (2007). Vitamin B12 deficiency in the aged: a population-based study. Age Ageing.

[B16] Zamboni V, Cesari M, Zuccala G, Onder G, Woodman RC, Maraldi C, Ranzini M, Volpato S, Pahor M, Bernabei R (2006). Anemia and cognitive performance in hospitalized older patients: results from the GIFA study. Int J Geriatr Psychiatry.

[B17] De Maria R, Ripamonti V, Sandri R, Ceretti AP, Ferratini M (2005). The negative prognostic synergism of anemia and heart disease in female nursing home residents. Am J Cardiol.

[B18] Penninx BW, Pluijm SM, Lips P, Woodman R, Miedema K, Guralnik JM, Deeg DJ (2005). Late-life anemia is associated with increased risk of recurrent falls. J Am Geriatr Soc.

[B19] Skjelbakken T, Langbakk B, Dahl IM, Lochen ML (2005). Haemoglobin and anaemia in a gender perspective: the Tromso Study. Eur J Haematol.

[B20] Wang JL, Shaw NS (2005). Iron status of the Taiwanese elderly: the prevalence of iron deficiency and elevated iron stores. Asia Pac J Clin Nutr.

[B21] Zakai NA, Katz R, Hirsch C, Shlipak MG, Chaves PH, Newman AB, Cushman M (2005). A prospective study of anemia status, hemoglobin concentration, and mortality in an elderly cohort: the Cardiovascular Health Study. Arch Intern Med.

[B22] Artz AS, Fergusson D, Drinka PJ, Gerald M, Gravenstein S, Lechich A, Silverstone F, Finnigan S, Janowski MC, McCamish MA, Ershler WB (2004). Prevalence of anemia in skilled-nursing home residents. Arch Gerontol Geriatr.

[B23] Choi CW, Lee J, Park KH, Yoon SY, Choi IK, Oh SC, Seo JH, Kim BS, Shin SW, Kim YH, Kim JS (2004). Prevalence and characteristics of anemia in the elderly: cross-sectional study of three urban Korean population samples. Am J Hematol.

[B24] Guralnik JM, Eisenstaedt RS, Ferrucci L, Klein HG, Woodman RC (2004). Prevalence of anemia in persons 65 years and older in the United States: evidence for a high rate of unexplained anemia. Blood.

[B25] Nandigam V, Nandigam K, Badhe BA, Dutta TK (2004). Is adult definition of anemia applicable to a geriatric population? Study of erythrocyte parameters in Indian geriatric inpatients. J Am Geriatr Soc.

[B26] Penninx BW, Pahor M, Cesari M, Corsi AM, Woodman RC, Bandinelli S, Guralnik JM, Ferrucci L (2004). Anemia is associated with disability and decreased physical performance and muscle strength in the elderly. J Am Geriatr Soc.

[B27] Semba RD, Guralnik JM, Chaves P, Ricks MO, Fried LP, Women's Health and Aging Studies (2004). Iron status and anemia in a population-based study of women with and without disability living in the community: the Women's Health and Aging Studies. Haematologica.

[B28] Coban E, Timuragaoglu A, Meric M (2003). Iron deficiency anaemia in the elderly: prevalence and endoscopic evaluation of the gastrointestinal tract in outpatients. Acta Haematol.

[B29] Fleming DJ, Jacques PF, Tucker KL, Massaro JM, D'Agostino RB, Wilson PW, Wood RJ (2001). Iron status of the free-living, elderly Framingham Heart Study cohort: an iron-replete population with a high prevalence of elevated iron stores. Am J Clin Nutr.

[B30] Mitrache C, Passweg JR, Libura J, Petrikkos L, Seiler WO, Gratwohl A, Stahelin HB, Tichelli A (2001). Anemia: an indicator for malnutrition in the elderly. Ann Hematol.

[B31] Olivares M, Hertrampf E, Capurro MT, Wegner D (2000). Prevalence of anemia in elderly subjects living at home: role of micronutrient deficiency and inflammation. Eur J Clin Nutr.

[B32] Spyckerelle Y, Piette F, Steinmetz J, Fournier B, Bussy C, Giordanella JP, Boulange M (2000). Iron deficiency in patients over 60 years. Descriptive study in the consultant population of health screening centers. Gastroenterol Clin Biol.

[B33] Izaks GJ, Westendorp RG, Knook DL (1999). The definition of anemia in older persons. JAMA.

[B34] Charlton KE, Kruger M, Labadarios D, Wolmarans P, Aronson I (1997). Iron, folate and vitamin B12 status of an elderly South African population. Eur J Clin Nutr.

[B35] Takasaki M, Tsurumi N, Konjiki O, Sakurai H, Kanou H, Yanagawa K, Katsunuma H (1997). Causes, diagnosis, and treatment of anemia in the elderly. Nippon Ronen Igakkai Zasshi.

[B36] Lesourd B, Decarli B, Dirren H (1996). Longitudinal changes in iron and protein status of elderly Europeans. SENECA Investigators. Eur J Clin Nutr.

[B37] Smieja MJ, Cook DJ, Hunt DL, Ali MA, Guyatt GH (1996). Recognizing and investigating iron-deficiency anemia in hospitalized elderly people. CMAJ.

[B38] Anía BJ, Suman VJ, Fairbanks VF, Melton LJ (1994). Prevalence of anemia in medical practice: community versus referral patients. Mayo Clin Proc.

[B39] Inelmen EM, D'Alessio M, Gatto MR, Baggio MB, Jimenez G, Bizzotto MG, Enzi G (1994). Descriptive analysis of the prevalence of anemia in a randomly selected sample of elderly people living at home: some results of an Italian multicentric study. Aging (Milano).

[B40] Cooper JW, Cobb HH (1992). Geriatric patient nutritional correlates and changes in a geriatric nursing home. J Geriatr Drug Ther.

[B41] Joosten E, Pelemans W, Hiele M, Noyen J, Verhaeghe R, Boogaerts MA (1992). Prevalence and causes of anaemia in a geriatric hospitalized population. Gerontology.

[B42] Salive ME, Cornoni-Huntley J, Guralnik JM, Phillips CL, Wallace RB, Ostfeld AM, Cohen HJ (1992). Anemia and hemoglobin levels in older persons: relationship with age, gender, and health status. J Am Geriatr Soc.

[B43] Kirkeby OJ, Fossum S, Risoe C (1991). Anaemia in elderly patients. Incidence and causes of low haemoglobin concentration in a city general practice. Scand J Prim Health Care.

[B44] Challand GS, Michaeloudis A, Watfa RR, Coles SJ, Macklin JL (1990). Distribution of haemoglobin in patients presenting to their general practitioner, and its correlation with serum ferritin. Ann Clin Biochem.

[B45] Woo J, Arumanayagam M, Ho SC, Swaminathan R (1989). Hematological indices and the prevalence of anemia in an elderly Chinese population. Pathology.

[B46] Nilsson-Ehle H, Jagenburg R, Landahl S, Svanborg A, Westin J (1988). Haematological abnormalities and reference intervals in the elderly. A cross-sectional comparative study of three urban Swedish population samples aged 70, 75 and 81 years. Acta Med Scand.

[B47] Celestin-Roux C, Hale WE, Perkins LL, Stewart RB (1987). Anemia: an evaluation of age, sex, disease and medications in a geriatric population. J Geriatr Drug Therap.

[B48] Timiras ML, Brownstein H (1987). Prevalence of anemia and correlation of hemoglobin with age in a geriatric screening clinic population. J Am Geriatr Soc.

[B49] Matilla KS, Kuuslea V, Pelliniemi TT, Rajamaki A, LaiHola HL, Juva K (1986). Haematological laboratory findings in the elderly: influence of age and sex. Scand J Clin Lab Invest.

[B50] Garry PJ, Goodwin JS, Hunt WC (1983). Iron status and anemia in the elderly: new findings and a review of previous studies. J Am Geriatr Soc.

[B51] Campbell AJ, Murphy C, Reinken J, Allan B (1981). Anaemia in old age: a study of prevalence and causes. N Z Med J.

[B52] Lipschitz DA, Mitchell CO, Thompson C (1981). The anemia of senescence. Am J Hematol.

[B53] Kalchthaler T, Tan ME (1980). Anemia in institutionalized elderly patients. J Am Geriatr Soc.

[B54] Patel KV, Harris TB, Faulhaber M, Angleman SB, Connelly S, Bauer DC, Kuller LH, Newman AB, Guralnik JM (2007). Racial variation in the relationship of anemia with mortality and mobility disability among older adults. Blood.

[B55] Herzog CA, Muster HA, Li S, Collins AJ (2004). Impact of congestive heart failure, chronic kidney disease, and anemia on survival in the Medicare population. J Card Fail.

[B56] van Dijk PT, Mehr DR, Ooms ME, Madsen R, Petroski G, Frijters DH, Pot AM, Ribbe MW (2005). Comorbidity and 1-year mortality risks in nursing home residents. J Am Geriatr Soc.

[B57] Wu WC, Rathore SS, Wang Y, Radford MJ, Krumholz HM (2001). Blood transfusion in elderly patients with acute myocardial infarction. N Engl J Med.

[B58] Beghe C, Wilson A, Ershler WB (2004). Prevalence and outcomes of anemia in geriatrics: a systematic review of the literature. Am J Med.

[B59] Wieczorowska-Tobis K, Mossakowska M, Niemir Z, Sawinski K, Klich-Raczka A, Zyczkowska J, Breborowicz A (2005). Hematologic parameters in Polish centenarians. Wiad Lek.

[B60] McClellan WM, Flanders WD, Langston RD, Jurkovitz C, Presley R (2002). Anemia and renal insufficiency are independent risk factors for death among patients with congestive heart failure admitted to community hospitals: a population-based study. J Am Soc Nephrol.

[B61] Langston RD, Presley R, Flanders WD, McClellan WM (2003). Renal insufficiency and anemia are independent risk factors for death among patients with acute myocardial infarction. Kidney Int.

[B62] Mozaffarian D, Nye R, Levy WC (2003). Anemia predicts mortality in severe heart failure: the prospective randomized amlodipine survival evaluation (PRAISE). J Am Coll Cardiol.

[B63] Komajda M, Anker SD, Charlesworth A, Okonko D, Metra M, Di Lenarda A, Remme W, Moullet C, Swedberg K, Cleland JG, Poole-Wilson PA (2006). The impact of new onset anaemia on morbidity and mortality in chronic heart failure: results from COMET. Eur Heart J.

[B64] Chaves PH, Carlson MC, Ferrucci L, Guralnik JM, Semba R, Fried LP (2006). Association between mild anemia and executive function impairment in community-dwelling older women: The Women's Health and Aging Study II. J Am Geriatr Soc.

[B65] Penninx BW, Pahor M, Woodman RC, Guralnik JM (2006). Anemia in old age is associated with increased mortality and hospitalization. J Gerontol A Biol Sci Med Sci.

[B66] Pan WH, Habicht JP (1991). The non-iron-deficiency-related difference in hemoglobin concentration distribution between blacks and whites and between men and women.. Am J Epidemiol.

[B67] Beutler E, West C (2005). Hematologic differences between African-Americans and whites: the roles of iron deficiency and alpha-thalassemia on hemoglobin levels and mean corpuscular volume. Blood.

[B68] Moore RA, Derry S, Makinson GT, McQuay HJ (2005). Tolerability and adverse events in clinical trials of celecoxib in osteoarthritis and rheumatoid arthritis: systematic review and meta-analysis of information from company clinical trial reports. Arthritis Res Ther.

