# Supplementary material for: Prevalence of anaemia in older persons: systematic review
Source: BMC Geriatr. 2008 Jan 14;8:1. doi: 10.1186/1471-2318-8-1 (PMC2248585; doi:10.1186/1471-2318-8-1)
Supplement: Additional file 1 — Electronic search strategy [file 1471-2318-8-1-S1.doc]

Search Strategy

MEDLINE (Ovid)

Anemia/ep with limits: “humans” and "all aged (65 and over)" and yr="1980 - 2007"

EMBASE (Ovid)

Anemia/ep with limits: “humans” and “aged <65+ years” and yr="1980 - 2007"
