# Supplementary material for: Prevalence of anaemia in older persons: systematic review
Source: BMC Geriatr. 2008 Jan 14;8:1. doi: 10.1186/1471-2318-8-1 (PMC2248585; doi:10.1186/1471-2318-8-1)
Supplement: Additional file 2 — Included studies. Details of studies included in the review [file 1471-2318-8-1-S2.pdf]

Additional file 2: Details of included studies

Reference	Study design and population	Number of patients	Anaemia definition	Associated pathology	Results	Community Nursing home Hospital	Comments
Atti et al.<br>Neurobiol Aging 2006 27:278-284<br>Sweden<br><br>**Kungsholmen Project**	Primary aim to evaluate role of anaemia on development of dementia<br>Longitudinal study (3y) of elderly non-demented subjects<br>Prevalence of anaemia at baseline reported<br>Community-based, non-demented<br>75-95 y	1377<br>Men 344<br>Women 1033	WHO definition and<br>Study defined<br>5th percentile (men Hb <117 g/L, women <116 g/L)<br>25th percentile (men Hb <135 g/L, women <129 g/L)	Excl if MMSE <20 at baseline, age >95 y, or unknown educational background	WHO<br>Overall 9.4%<br>Men 15.7%<br>Women 7.3%<br><br>5th percentile<br>Overall 4.9%<br>25th percentile<br>Overall 24.9%	Community	At 3y anaemia associated with dementia for subjects with good cognitive function at baseline, but not sig for those with cognitive impairment at baseline
Culleton et al.<br>Blood 2006 107:3841-3846<br>Canada	Primary aim of study to determine correlation between anaemia and hospital admission or mortality<br>Prevalence of anaemia reported<br>Individuals independent in community with Hb and creatinine measured<br>>65 y	17030<br>Women 9471<br>Men 7559<br>Mean age 75 y	WHO definition and<br>Hb ≤110 g/L	No restrictions for co-morbidity<br>Exclusions were: died within 30 days, and early dialysis	WHO<br>Overall 13%<br>Men 14.3%<br>Women 12.0%<br>66-70y 8.7%<br>71-75y 10.9%<br>76-80y 14.2%<br>81-85y 20.4%<br>>85y 23.0%<br><br>Hb <110 g/L<br>Overall 4.2%<br>66-70y 2.7%<br>71-75y 3.0%<br>76-80y 4.8%<br>81-85y 7.5%<br>>85y 7.5%	Community	Criterion of measured Hb and creatinine for inclusion could exclude healthiest people<br>Anaemia associated with increased risk of hospital admission and mortality, with higher mortality at lower Hb, with hazard ratios from 1.5 to 4 as Hb fell from 130 to <110 g/L<br>Anaemia more common in patients with diabetes and/or low GFR
Denny et al.<br>Am J Med 2006 119:327-334<br>USA<br><br>(**DUKE) EPESE study**	Primary aim of study to determine relationship between Hb and mortality, cognition and function in elderly<br>Prevalence of anaemia reported<br>Prospective cohort study<br>Household sample<br>>70 years	1744<br>Men 610<br>Women 1134<br>Mean age 78 y	WHO definition	Excluded those unable/unwilling to give blood sample or unable to give informed consent	Overall 24%<br>Men 24%<br>Women 24%<br>65-74y 17%<br>75-79y 25%<br>>80y 32%	Community	African-Americans oversampled (54%)<br>No difference between sexes<br>African-Americans 3x more likely to be anaemic (17.6%) than Caucasians (7%)<br>Anaemia associated with inc risk of mortality over 8 y<br>Strong association with poor physical function and cognitive function., and predictive of decline
Dharmarajan et al.<br>J Am Med Dir Assoc 2006 7:287-93	Primary aim of study to investigate whether anaemia is associated with falls in elderly patients during hospitalisation for acute care unrelated to a fall.<br>Patients admitted from community and nursing homes.	362<br>Men 166<br>Women 196<br>Mean age 77 y<br>(59-104 y)	WHO definition	None specified	Overall<br>Men 52%<br>Women 42%	Hospital	No significant differences between patients from nursing homes and community<br>Patients who fell were sig more likely to be anaemic

Hammerman-Rozenberg et al.<br>Age Ageing 2006 35:514-517<br>Israel<br><br>**Jerusalem Longitudinal Study**	Primary aim of to study to identify relationship between low dose aspirin and anaemia in 77 y-olds<br>Prevalence of anaemia reported<br>Cross-sectional study using representative cohort of community dwelling individuals	464<br>Men 237<br>Women 227<br>age 77 y	WHO definition	Chronic low dose aspirin used by 29% women and 38% men.<br>Most took 100 mg/day	Age 77 y<br>Overall 17%<br>Men 18.6%<br>Women 15.4%<br><br>Prevalence sig lower in regular users of aspirin	Community	No details of how studied population was selected: "representative of community dwelling"
Joosten et al.<br>Gerontology<br>2006 52: 382-385<br>Belgium	Primary aim of study to investigate whether anaemia is risk factor for delirium<br>Prevalence of anaemia within 48h of admission reported<br>Hospitalised geriatric population<br>≥70 y	190<br>Men 68<br>Women 122<br>Mean age 83 y	WHO definition	Excluded if had had red cell transfusion in preceding 3 months<br>All pts had MMSE-12 evaluation in previous study<br>Clinical diagnosis of: infectious disorder in 37%,<br>cardio-respiratory disorder in 20%,<br>neuropsychiatric disorder in 17%	Overall 50%<br>Men 53%<br>Women 48%	Hospital	Claims sig association for anaemia and delirium for men, not women, but small numbers and wide CIs
Loikas et al.<br>Age Ageing 2007 36:177-83, e-pub Dec 2006<br>Finland	Primary aim to study vit B12 deficiency in elderly<br>Cross-sectional study of whole population ≥65 y (66% participated, 64% had results for anaemia)<br>6% in long-term institution	1021<br>Men 423<br>Women 598<br><br>Median age 73 y	Hb <134 g/L (men), <117 g/L (women)	No details of those who did not participate.<br>Those without data for anaemia had vit B12 deficiency diagnosed, and were older and more likely to be institutionalised	Overall 13.4%<br>Men 21.5%<br>Women 7.7%	Community (6% in long-term institution)	May underestimate prev due to excl of vit B12 deficient individuals.<br>Some details of medications
Zamboni et al.<br>Int J Geriatric Psychiatry 2006 21:529-34<br>Italy<br><br>**GIFA study**	Primary aim of study to determine correlation of Hb and cognitive function on admission to hospital<br>Cross-sectional, consecutive patients admitted in 1993, 1995, 1997<br>Prevalence of anaemia reported	13301<br>Men 6651<br>Women 6650<br>Mean age 72 y	WHO definition	Excluded those with missing values for Hb or cognitive function	Overall 39.6%	Hospital	May include some patients <65 y, and potential for some patients to be counted >once if readmitted<br>Some details on medical therapy<br>Anaemia more common in patients with cognitive impairment, even when adjusted for age
De Maria et al.<br>Am J Cardiol 2005 96:1460-1462<br>Italy	Primary aim of study to investigate prognostic synergism between anaemia and heart disease in nursing home residents<br>Prevalence of anaemia reported<br>(61-104 y)	441<br>All women<br>Mean age 87 y	WHO definition and Hb ≤110 g/L	Renal function normal in 10.5%, insufficient in 33.5%	WHO<br>Women 49.7%<br><br>Hb ≤110 g/L<br>Women 24.5%	Nursing home	2/3 > 85 y<br>No details of how sample obtained<br>Impaired renal function signif assoc with anaemia<br>1 y mortality inc x3 if anaemia and heart disease present

Penninx et al.<br>JAGS 2005 53:2106-2111<br>Netherlands<br><br>**Longitudinal Aging Study Amsterdam**	Primary aim of study to examine if anemia is associated with recurrent falls<br>Random sample of primarily community living individuals from one of three regions in main study<br>Prevalence of anaemia reported<br>≥65 y	394<br>Men 196<br>Women 198<br>Mean age 75 y	WHO definition	None reported here	Overall 11.9%<br>Men 14.9%<br>Women 9.1%	Community	Individuals who did not have Hb measurement were older (3 y)<br>Anaemic individuals were older, had lower BMI, higher creatinine, more comorbidity<br>Anaemia associated with falls
Skjelbakken et al. Eur J Haematol 2005 74:381-388<br>Norway<br><br>**Tromso Study**	Primary aim of study to determine distribution of anaemia<br>Cross-sectional study<br>Total birth cohort (77% participated)<br>≥25 y, with subgroup ≥65 y	4228<br>Men 1785<br>Women 2443	WHO definition and<br>2.5% percentile (Hb <129 g/L men, <114 g/L women), and also<br>men Hb <100 g/L, women <90 g/L		WHO criteria<br>Overall 7.1%<br>Men 10%<br>Women 5%<br>65-74y 4.9%<br>75-84y 10.0%<br>85+y 21%<br><br>men <110 g/L, women <90 g/L<br>Overall 0.2%	Community	
Wang and Shaw.<br>Asia Pac J Clin Nutr 2005 14:278-284<br>Taiwan<br><br>**Elderly NAHSIT study**	Primary aim of study to assess iron status in elderly population<br>Nationally representative cross-sectional sample of community ("free") living individuals<br>Prevalence of anaemia reported<br>≥65 y	2354<br>Men 1202<br>Women 1152	WHO definition	Excluded if institutionalised	Overall 18.8%<br>Men 18.7%<br>Women 18.9%	Community	Serum ferritin levels sig lower in subgroup with history of GI ulcer than without<br>Other iron indices not associated with ulcer
Zakai et al.<br>Arch Intern Med 2005 165:2214-2220<br>USA<br><br>**Cardiovascular Health Study**	Primary aim of study to determine relationship between Hb level and mortality<br>Prospective cohort study of community dwelling individuals<br>Prevalence of anaemia reported<br>≥65 y	5797<br>Men 2466<br>Women 3331<br>Mean age 73 y	WHO definition	Excluded if institutionalised, wheelchair bound, being treated for cancer, unable to give informed consent	Overall 8.5%<br>Men 9.2%<br>Women 8.1%	Community	Prevalence higher in black (17.6%) than white (7.0%) individuals, and those with more comorbidity<br>Low Hb associated with low BMI and activity level, poor self-reported health, frailty, CHF, stroke, TIA<br>Inc risk of mortality over 11 y (at both extremes of Hb)
Artz et al.<br>Arch Gerontol Geriatr 2004 39:201-206<br>USA	Primary aim of study to determine prevalence of anaemia and association with hospitalisation in chronic care residents in skilled nursing homes<br>Modified cross-sectional study (chart review over 6 month period)<br>87% 65y or more	900<br>Men 336<br>Women 564<br>Mean age 79 y, median age 82 y	WHO definition and<br>Hb <100 g/dL	Excluded if acute rehabilitation resident or "inappropriate for participation"	WHO<br>Overall 48%<br>Men 47%<br>Women 49%<br><br>Hb <100 g/L<br>Overall 11.4%	Nursing home	Chart review over 6 months may over-estimate prevalence of anaemia<br>Anaemia associated with x2 rate of hospitalisation (30 vs 15%)<br>Residents with Hb<10g/dL had 55% hospitalisation rate
Choi et al.<br>Am J Haematol<br>2004 77:26-30<br>Korea	Primary aim to study prevalence and characteristics of anaemia<br>Cross sectional survey<br>Randomly selected sample of "apparently healthy" community based individuals from urban population<br>60-95 y	1254<br>Men 273<br>Women 981<br>Median age 70 y	WHO definition		Overall 13.6%<br>Men 9.9%<br>Women 14.7%<br>60-69 y 7.6%<br>70-79 y 16.5%<br>≥80 y 25.4%	Community	Causes of anaemia also investigated<br><br>Logistic regression identified independent risk factors for anaemia to be female sex, older age, lower BMI, higher creatinine level, lower albumin level

Guralnik et al.<br>Blood 2004 104: 2263-2268<br>USA<br><br>**NHANES III**	Primary aim was population study (children and adults, civilian, non-institutionailzed)<br>Stratified sampling<br>Sub-group analysis for ≥65 y (oversampling of African Americans and Mexican Americans in this group)	4199 (≥65 y)	WHO definition and<br>Hb <110 g/L	Patients not institutionalised	WHO<br>Overall 10.6%<br>Men 11%<br>Women 10.2%<br>65-74 y 8%<br>75-84 y 13%<br>≥85 y 23%<br><br>Hb<110 g/L<br>Men 1.6%<br>Women 2.8%	Community	Anaemia increasingly prevalent with increasing age, usually mild<br>3x more common in non-Hispanic blacks (27.8%) than non-Hispanic whites (9.0%) and Mexican Americans (10.4%)<br><br>Data on causes of anaemia presented
Nandigam and Nandigam.<br>JAGS 2004 52:1589-1560<br>India	Case control study of erythrocyte parameters in consecutive patients admitted to hospital for any cause<br>Prevalence of anaemia in cohort of elderly reported<br>≥65 y	100<br>Men 37<br>Women 63<br>mean age 70 y	WHO definition and<br>Study defined (Hb <120 g/L for men, 115 g/L for women)		WHO<br>Overall 72%<br><br>Study defined<br>Overall 61%	Hospital	
Penninx et al.<br>JAGS 2004 52:719-724<br>Italy<br><br>**InCHIANTI**	Primary aim of study to determine correlation between anaemia and disability, physical performance and muscle strength<br>Stratified random sample from urban population<br>Community dwelling<br>Prevalence of anaemia reported<br>≥65 y	1008<br>Men 444<br>Women 564<br>Mean age 75 y	WHO definition	Not reported here	Overall 11.3%<br>Men 11.1%<br>Women 11.5%	Community	Anaemia associated with more disabilities, lower physical performance, lower muscle strength, also lower body mass index, lower cognitive function, lower creatinine, presence or history of various diseases
Semba et al.<br>Haematologica 2004 89:357-358<br>USA<br><br>**Women Health and Aging Study I and II**	Primary aim of study to investigate iron status and its relation to disability<br>Age-stratified random sample of community-dwelling women representing the one-third most disabled and two-thirds least disabled<br>Community dwelling<br>Prevalence of anaemia reported<br>70-79 y	679<br>All women	WHO definition	MMSE ≥18	Women 13.3%	Community	
Coban et al.<br>Acta Haematol 2003 110:25-28<br>Turkey	Primary aim of study to determine prevalence of iron-deficiency anaemia in elderly people<br>Referrals to secondary-care out-patient clinics<br>Cross-sectional study<br>Prevalence of anaemia reported<br>>65 y	1388	WHO definition	None described	Overall 25%	Community	
Fleming et al.<br>Am J Clin Nutr 2001 73:638-646<br>USA<br><br>**Framingham Heart Study**	Primary aim of study to determine iron status of elderly people<br>Framingham Heart Study originally random selection from census list. This study on surviving members after 40 y<br>Cross-sectional study<br>67-96 y	1016<br>Men 411<br>Women 605<br>Mean age 76 y	WHO definition by Hct <39% men, <36% women and<br>NHANES III definition Hb<124 g/L men, Hb<118 g/L women	All whites<br>Required adequate serum to determine CRP and iron indices	WHO<br>Overall 16.7%<br>Men 16.0%<br>Women 17.2%<br><br>NHANES III<br>Overall 8.7%<br>Men 6.1%<br>Women 10.5%	Community	

Mitrache et al.<br>Ann Hematol<br>2001 80:295-298<br>Switzerland	Primary aim of study to assess prevalence of anaemia and relate this to nutritional status<br>Consecutive patients admitted to geriatric unit in 1997	186<br>Men 93<br>Women 93<br>Median age 85 y<br>(range 56-100 y, four patients <65 y)	Hb <120 g/L	Four patients younger than 65 y, but had conditions typical for geriatric population	Overall 44.1%<br>Men 43.0%<br>Women 45.2%	Hospital	
Olivares et al.<br>Eur J Clin Nutr 2000 54:834-839<br>Chile	Primary aim of study to determine prevalence of anaemia and association with diet<br>Cross-sectional study<br>Urban community, "apparently healthy" from low socio-economic level district	274<br>Men 93<br>Women 181<br>Mean age 70 y	WHO definition	Excluded if had any acute or chronic disease, or hospital admission or treatment with any drug or vitamin etc likely to influence haematopoeisis in preceding 6 months	Overall 4.7%<br>Men 5.4%<br>Women 4.4%	Community	Prevalence of anaemia, abnormal iron status, low serum copper and deficient vit B12 very low in both sexes<br>Prevalence of anaemia higher with inflammation (men 22%, women 32%)<br>Hb decreased with age
Spyckerelle et al.<br>Gastroenterol Clin Biol 2000 24:709-713<br>France	Primary aim of study to determine frequency of iron deficiency in elderly<br>Population undergoing a periodic "Health screening examination" for sickness insurance<br>Prevalence of anaemia reported<br>60-75 y	6644<br>Men 3524<br>Women 3120	WHO definition	Excluded if had blood transfusion within preceding 3 months, current or recent use of dietary supplements<br><br>Over 70 y under-represented. Some sectors of society not included	Overall 3.3%	Community	
Izaks et al.<br>JAMA 1999 281:1714-1717<br>Netherlands<br><br>**Leiden 85+ Study**	Primary aim to determine correlation between Hb and cause-specific mortality in elderly in 10 y prospective study<br>Community-based (home or nursing home)<br>Prevalence of anaemia at baseline reported<br>≥85 y	755<br>Men 211<br>Women 544<br>Median age 89 y	WHO definition	No exclusion criteria<br>Hb levels available for 74% of eligible sample	Overall 20%<br>Men 28%<br>Women 17%	Community (some in nursing home)	Regression analysis showed association between anaemia and increased mortality (higher mortality with lower Hb) over 5 y, but not for 5-10 y<br>Anaemia associated with disease (malignant neoplasm, peptic ulcer, infection)
Charlton et al.<br>Eur J Clin Nutr 1997 51:424-430<br>South Africa	Aim to determine prevalence of anaemia and haemopoietic nutrient status in elderly of mixed ancestry<br>Random sample, urban, non-institutionalised<br>≥65 y	187<br>Men 88<br>Women 99<br>Mean age 74 y	WHO definition	Low income group<br>Excluded if had cognitive impairment based on minimal screening using three questions	Overall 13.9%<br>Men 11.4%<br>Women 16.2%	Community	Mean energy intake sig < RDA<br>1/3 of anaemic individuals had haemopoeitic nutrient deficiencies, including iron
Takasaki et al.<br>Jpn J Geriatr<br>1997 34:171-179<br>Japan	Aim of study to investigate the causes, diagnosis and treatment of anaemia in the elderly<br>Subjects undergoing medical examination at hospital geriatric outpatient dept but described as "healthy"<br>≥65 y	3583<br>Men 1590<br>Women 1993	Hb ≤110 g/L	No details	Overall 13%	Community	Abstract only (paper In Japanese)<br>Mean Hb and Hct lower in older age groups

Lesourd et al.<br>Eur J Clin Nutr 1996 50 (Suppl2):S16-S24<br>Europe<br><br>**SENECA**	Primary aim of study to determine iron and protein status in elderly<br>Community longitudinal study of free-living elderly subjects<br>Prevalence of anaemia reported at baseline	1921<br>Men 975<br>Women 946<br>age 70-75 y	WHO definition and NHANES II definition (Hb<126 g/L men, <117 g/L women)	No details here	WHO<br>Overall 5.6%<br>Men 5.6%<br>Women 5.5%<br><br>NHANES II<br>4.2%	Community	
Smieja et al.<br>Can Med Assoc J 1996 155:691-696<br>Canada	Primary aim of study to determine whether anaemia is appropriately documented and investigated for iron deficiency<br>Consecutive medical admissions to secondary care ≥65<br>Prevalence of anaemia reported	183<br>Men/Women unknown<br>Mean age 75 y	Hb checked on 2 occasions, on both -<br>Hb <120 g/L men<br>Hb <110 g/L women	Excluded if ≥72 hours after admission or discharged transferred or died within 48 hours	Overall 36%	Hospital	
Ania et al.<br>Mayo Clin Proc 1994 69:730-735<br>USA<br><br>**Olmsted county**	Primary aim of study to compare prevalence of anaemia in the community with referrals to secondary care<br>Cross-sectional study<br>Subgroup ≥65 y (92% of eligible population in community)	N of referral patients not given<br>Olmsted (community) patients 9149 (≥65 y)<br>Men 3436<br>Women 5713	WHO definition	No exclusion criteria described<br>Population 95% Caucasian	Olmsted patients: 17.6%<br>Men 20.5%<br>Women 15.9%<br><br>Insufficient data to analyse referral patients	Community	At least 1 in 4 individuals with Hb ≤110g/L did not have anaemia recorded as diagnosis in medical record
Inelmen et al.<br>Aging (Milano) 1994 6:81-89<br>Italy<br><br>**VENSE + INESE**	Primary aim of study to determine hematological characteristics of an elderly population<br>Cross-sectional study of community-based, apparently healthy individuals<br>Prevalence of anaemia reported<br>≥65 y	1784<br>Men 725<br>Women 1059	WHO definition and Hb ≤120 g/L	None described but patients were not institutionalized	WHO<br>Overall 9%<br>Men 9.4%<br>Women 8.8%<br><br>Hb ≤120 /L<br>Overall 7.1%<br>Men 2.9%<br>Women 9.9%	Community	Prevalence of anaemia (WHO) increased with age for all age groups for women, and 70+ for men
Cooper et al.<br>J Geriatric Drug Ther 1992 6:73-82<br>USA	Aim to determine nutritional correlates and changes in nursing home residents<br>Chart review of patients in long-term care<br>Prevalence of anaemia on admission reported	175<br>Men 49<br>Women 126<br>Mean age 81 y	not explicitly stated in abstract, probably Hb <120 g/L		Overall 24%	Nursing home	Abstract only
Joosten et al.<br>Gerontology 1992 38:111-117<br>Belgium	Primary aim of study to determine prevalence and causes of anaemia<br>Consecutive admissions to acute geriatric hospital over 6-month period<br>≥65 y	732<br>Men 289<br>Women 443<br>Median age 81 y	Hb ≤115 g/L<br>Hb 100-114g/dL<br>Hb <80g/dL	No exclusion criteria described.	Hb ≤115g/dL<br>Overall 24%<br>Hb 100-114 g/dL<br>Overall 14%<br>Hb <80g/dL<br>Overall 3.1%	Hospital	Causes of anaemia identified in 83% cases, mainly anaemia of chronic disease (35%) and iron deficiency anaemia (15%)
Salive et al.<br>JAGS 1992 40:489-496<br>USA<br><br>**EPESE (E Boston, Iowa, New Haven)**	Primary aim of study to determine relationship between haemoglobin and anaemia with age, gender, health status<br>Cross-sectional, community study<br>Prevalence of anaemia reported<br>≥71 y	3946<br>Men 1406<br>Women 2540	WHO definition	No exclusion criteria described	Overall 13.5%<br>Men 15.2%<br>Women 12.6%<br>71-74y 8.6%<br>75-79y 12.4%<br>80-84y 15.3%<br>85+y 22.4%	Community	Lower Hb independently associated with older age, black race, low BMI, diagnosis and hospitalisation in previous year for cancer

Kirkeby et al.<br>Scand J Prim Health Care 1991 9:167-171<br>Norway	Primary aim of study to determine prevalence of anaemia and its causes<br>Community, patients attending primary care service<br>>70 y	530<br>Men 108<br>Women 422<br>Mean age 79 y	Hb <133 gL (men)<br>Hb <120 g/L (women)	No exclusion criteria	Overall 14%<br>Men 16%<br>Women 13%	Community	Causes of anaemia investigated<br>Prevalence of anaemia possibly overestimated due to some retesting
Challand et al.<br>Ann Clin Biochem 1990 27(Pt 1):15-20<br>UK	Primary aim of study to determine prevalence of anaemia and iron deficiency in elderly, and in younger women, presenting to doctor in primary care (GP)<br>Community<br>≥65 y	307<br>Men 154<br>Women 153	WHO definition	No exclusion criteria but attended GP surgery<br>Not taking iron	Overall 16.9%<br>Men 20.1%<br>Women 13.7%	Community	Caucasian
Woo et al.<br>Pathology 1989 21:31-34<br>Hong Kong	Primary aim of study to determine reference ranges for haematological indices and assess prevalence anaemia in the elderly<br>Community, lower socio-economic group, living independently in social housing<br>≥ 60 y	427<br>Men 171<br>Women 256<br>Mean age 70 y	WHO definition	Excluded if had any diseases causing anaemia, haemoglobinopathies, any blood test value ±3SD from mean value	Overall 9.6%<br>Men 10.5%<br>Women 9.0%	Community	Population capable of self care and normal active life, but low income, so may not be representative of general population
Nilsson-Ehle et al.<br>Acta Med Scand 1988 224:595-604<br>Sweden<br><br>**70 y-old people in Gothenburg Study**	Primary aim of study to determine haematological values and hence prevalence of anaemia and reference values<br>Representative sample of 70, 75 and 81 y-olds in Gothenburg<br>Baseline prevalence of anaemia reported	312 (age 70 y)<br>Men 148<br>Women 164<br><br>486 (age 75 y)<br>Men 205<br>Women 281<br><br>404 (age 81 y)<br>Men 145<br>Women 259	WHO definition and population-defined percentiles		WHO<br>70 y 4.8%<br>Men 5.4%<br>Women 4.2%<br><br>75 y 4.5%<br>Men 6.3%<br>Women 3.2%<br><br>81 y 10.4%<br>Men 13.1%<br>Women 8.9%	Community	Used for age distribution only
Celestin-Roux et al.<br>J Geriatr Drug Ther 1987 1:63-86<br>USA<br><br>**Dunedin Program (Florida)**	Primary aim of study to determine incidence, prevalence, risk factors for anaemia.<br>Community-based, longitudinal study with baseline prevalence of anaemia reported<br>≥65 y	3299<br>Men 1267<br>Women 2032	Hb <140 g/L(men)<br>Hb <120 g/L(women)	No exclusion criteria stated	Overall 25.3%<br>65-69 y<br>Men 53.4%<br>Women 10.7%<br>70-74 y<br>Men 31.6%<br>Women 7.9%<br>75-79 y<br>Men 52.3%<br>Women 16.9%<br>80-84 y<br>Men 55.5%<br>Women 19.4%<br>≥85 y<br>Men 25%<br>Women 13%	Community	Men had consistently higher levels of anaemia than women (3-5x), probably because of cut off used for men in this study<br>Decrease in Hb with age in both men and women, but more marked in men<br><br>Number of medical disorders not sig related to presence of anaemia<br>Inverse relationship between Hb and number of drugs used. No sig associations with particular drugs after adjusting for age

Timiras and Brownstein<br>J Am Geriatr Soc 1987<br>35:639-643<br>USA	Primary aim of study to determine prevalence of anaemia and correlation with age<br>Retrospective study of patients attending geriatric screening clinic<br>Community-based<br>≥60 y	1024<br>Men 395<br>Women 629<br>Mean age 70 y	Hb <140 g/L or Hct <40% (men)<br>Hb <120 g/L or Hct <37% (women)	Excluded if not ambulant or have seen a physician in preceding year	Overall 12%<br>Men 17.7%<br>Women 8.4%<br>60-64y 8.7%<br>65-69y 10.6%<br>70-74y 12.0%<br>75-79y 12.5%<br>≥80 y 23%	Community	Authors argue that Hb does NOT change with age but that there is a sex difference. May be due to choice of criteria for anaemia
Mattila et al.<br>Scan J Clin Lab Invest<br>1986 46:411-415<br>Finland	Primary aim of study to determine changes in haematological tests with age<br>Population study with 72% participation, included patients in residential homes and hospitals, but most were "relatively fit", with "most" living at home<br>Prevalence of anaemia reported<br>≥65 y	340<br>Men 180<br>Women 160	WHO definition<br>and<br>Hb <120 g/L (men), <115 g/L (women)	None described	WHO<br>Overall 7.6%<br>Men 9.4%<br>Women 5.6%	Community (some in homes or hospital)	
Garry et al.<br>J Am Geriatr Soc 1983<br>31:389-399<br>USA	Primary aim of study to determine iron status using various blood components including Hb<br>Prevalence of "low" Hb noted<br>Free-living, "healthy"<br>>60 y	280<br>Men 131<br>Women 149<br>median age 72 y	"low" Hb<br>Men <140 g/L<br>Women <120 g/L	Excluded if had known "serious medical conditions" or taking prescription medication	Men 2.3%<br>Women 0%	Community	White (3% Hispanic)<br>Exclusion of individuals taking any prescription medication probably accounts for low prevalence of anaemia
Campbell et al.<br>NZ Med J 1981 94:209-211<br>New Zealand	Primary aim of study to determine prevalence and causes of anaemia<br>Random sample of population living in own home, residential homes, or in hospital<br>≥65 y	557<br>Men 196<br>Women 361	Hb <120 g/L<br>Hb <110 g/L		Hb <120 g/L<br>Overall 10.8%<br>Men 4.0%<br>Women 14.4%<br><br>Hb <110 g/L<br>Overall 3.4%	Community (some in homes or hospital)	Dietary intake same in anaemic and non-anaemic individuals<br>In women >80 y, Hb dropped significantly with age<br><br>No sig diff between those taking NSAIDs and those not, except for men aged 65-74 y, who had sig lower Hb (note small numbers)
Lipschitz et al.<br>Am J Haematol 1981<br>11:47-54<br>USA	Primary aim of study to determine prevalence of anaemia in apparently well elderly people, and to document aetiology and evaluate significance<br>Individuals attending community activities for the elderly. Economically deprived community<br>>65 y	222<br>Men 26<br>Women 196<br>mean age 75 y	Hct <40% (men), <36% (women)	Excluded if had admission to hospital in previous year, cancer, inflammatory disorder or chronic disease possibly associated with anaemia, or were taking medication known to affect marrow function (included drugs for diabetes, heart failure, hypertension)	Overall 26%<br>Men 34%<br>Women 21.4%	Community	Black 105, white 117<br>Anaemia more prevalent in blacks than whites (x3)
Kalchthaler and Tan.<br>J Am Geriatr Soc<br>1980 28: 108-113<br>USA	Primary aim of study to evaluate anaemia in institutionalised elderly patients<br>Retrospective study of nursing home patients<br>Baseline prevalence of anaemia reported<br>Small number of patients were 41-60 y	161<br>140 over 60 y	WHO definition	Excluded if did not have full blood count, urinalysis, fecal blood, folate, B12, iron and iron-binding capacity	Overall 31.4 %	Nursing home	
