# Supplementary material for: Prevalence of anaemia in older persons: systematic review
Source: BMC Geriatr. 2008 Jan 14;8:1. doi: 10.1186/1471-2318-8-1 (PMC2248585; doi:10.1186/1471-2318-8-1)
Supplement: Additional file 3 — Excluded studies. Citation and reason for exclusion [file 1471-2318-8-1-S3.pdf]

Additional file 3: Excluded studies

Reference	Reason for Exclusion
Muzzarelli and Pfisterer. Am Heart J Cardiol 2006 152:991-996	Specific disease group
Penninx et al. J Gerontology 2006 61A:474-479	EPESE - use Salive 1992
Semba et al. Biological Trace Element Research 2006 112:97-108	Women's Health and Aging Studies I and II - use Semba 2004
Beutler and West. Blood 2005 106:740-745	Data for elderly not given separately
Cesari et al. Osteoporosis Int 2005 16:691-699	in Chianti - use Penninx 2004
Chaves et al J Gerontol A Biol Sci Med Sci 2005 60A:729-735	Women's Health and Aging Studies I and II - use Semba 2004
Choi et al. Arch Gerontol Geriatr 2005 41:303-309	Incidence study, and is part of Choi 2004
Ershler et al. JAGS 2005 53:1360-1365	Not prevalence study, not limited to elderly
Hollowell et al. Vital Health Stat 2005 11:1-156	NHANESIII No data on anaemia - use Guralnik 2004
Ischine et al. JAGS 2005 53:733-734	No age limits given
van Dijk PT et al. JAGS 2005 53:660-665	No definition of anaemia given
Wieczorowska-Tobis K et al. Wiadomosci Lekarskie 2005 58:56-61	<100 subjects
Cesari et al. J Gerontol A Biol Med Sci 2004 59:249-254	In Chianti - use Penninx 2004
Chaves et al. JAGS 2004 52:1811-1816	Women's Health and Aging study I - use Semba 2004
Cheng et al. Lab Hematol 2004 10:42-53	NHANES III. No useable data - use Semba 2004
Herzog et al. J Cardiac Fail 2004;10:467-472	No definition of anaemia - uses ICD code
Penninx et al. Am J Med 2003 115:104-110	EPESE (Iowa and Washington state) - use Salive 1992
Yamada et al. Eur J Haematol 2003 70:129-135	No prevalence data
Chaves et al. JAGS 2002 50:1257-1264	Women's Health and Aging studies I and II - no prevalence data - use Semba 2004
Cherentsky et al. Harefuah 2002 141:591-594	In Hebrew, translation not available
Argyriadou et al. BMC Family Practice 2001 2:5	Two subgroups <100 subjects
Fischbacher et al. BMJ 2001 322:958-959	No separate data for elderly

Kikuchi et al. JAGS 2001 49:1226-1228	No prevalence data
Wu et al. N Engl J Med 2001 345:1230-1236	Complete cohort not adequately described. Single threshold for men and women
Nilsson-Ehle et al. Eur J Haematol 2000 65:297-305	70 y-old people in Gothenburg study - use Nilsson-Ehle 1988
Ania et al. Am J Geriatr Soc 1997 45:825-831	Olmsted county - use Ania 1994
Lombardi et al. JAGS 1996 44:740-741	Not prevalence study
Liu et al. Gaoxiong Yi Xue Ke Xue Za Zhi 1994 10:683-688	In Chinese, translation not available
Ohhara et al. Nippon Ronen Igakkai Zasshi 1994 31:548-553	In Japanese, translation not available
Kafatos et al. J Am Coll Nutr 1993 12:685-692	Abstract only available - insufficient information
Dirren et al .Eur J Clin Nutr 1991 45(Suppl3):43-52	SENECA - use Lesourd 1996
Chen et al. Int J Vitam Nutr Res 1989 59:207-213	Two subgroups <100 subjects
Zauber and Zauber. JAMA 1987 257:2181-2184	<100 subjects
Dallman et al. Am J Clin Nutr 1984 39:437-445	NHANES II, use Guralnik 2004 (NHANES III) - may be overalp, study similar populations
Yip et al. Am J Clin Nutr 1984 39:427-436	NHANES II. No prevalence data
Hale et al. Age Ageing 1983 12:275-284	Dunedin Program - use Celestin-Roux 1987
Landahl et al. Clin Chim Acta 1981 112:301-314	70 y-old people in Gothenburg study - use Nilsson-Ehle 1988
Jernigan et al. J Am Geriatr Soc 1980 28:308-314	<100 subjects
